# MRI Assessment of Cardiac Function and Morphology in Adult Patients With Growth Hormone Deficiency: A Systematic Review and Meta-Analysis

**DOI:** 10.3389/fendo.2022.910575

**Published:** 2022-06-10

**Authors:** Fabio Bioletto, Nunzia Prencipe, Alessandro Maria Berton, Chiara Bona, Mirko Parasiliti-Caprino, Riccardo Faletti, Ezio Ghigo, Silvia Grottoli, Valentina Gasco

**Affiliations:** ^1^Endocrinology, Diabetology and Metabolism, Department of Medical Sciences, University of Turin, Turin, Italy; ^2^Radiology Unit, Department of Surgical Sciences, University of Turin, Turin, Italy

**Keywords:** cardiovascular system, cardiac magnetic resonance imaging, growth hormone deficiency, growth hormone replacement therapy, meta-analysis

## Abstract

**Background:**

Adult GH deficiency (GHD) has been described as a heterogeneous condition characterized by many clinical modifications, such as metabolic alterations, impaired quality of life, and increased mortality. The clinical relevance of cardiac involvement remains, however, only partially elucidated.

**Methods:**

PubMed/Medline, EMBASE, Cochrane library, OVID and CINAHL databases were systematically searched until February 2022 for studies evaluating cardiac function and morphology by magnetic resonance imaging in adult patients with GHD. Effect sizes were pooled through a random-effect model.

**Results:**

Four studies were considered in the meta-analysis. With respect to the left ventricle, GHD patients were characterized by a lower stroke-volume-index (-3.6 ml/m^2^, standardized mean difference (SMD) -0.60, 95%CI [-1.15,-0.05], p=0.03), lower end-diastolic-volume-index (-6.2 ml/m^2^, SMD -0.54, 95%CI [-0.97,-0.10], p=0.02) and, after accounting for possible biases, lower mass-index (-15.0 g/m^2^, SMD -1.03, 95%CI [-1.89,-0.16], p=0.02). With respect to the right ventricle, a lower end-diastolic-volume-index (-16.6 ml/m^2^, SMD -1.04, 95%CI [-2.04,-0.03], p=0.04) and a borderline-significant lower stroke-volume-index (-5.0 ml/m^2^, SMD -0.84, 95%CI [-1.77,0.08], p=0.07) could be observed. Data about the effect of GH replacement therapy highlighted a significant increase in left ventricular mass-index after treatment initiation (+3.7 g/m^2^, 95%CI [1.6,5.7], p<0.01).

**Conclusion:**

With respect to the left ventricle, our results confirmed those retrieved by echocardiographic studies. In addition, significant alterations were demonstrated also for the right ventricle, for which echocardiographic data are nearly absent. This supports the thesis of a biventricular cardiac involvement in patients with GHD, with a similar pattern of morphological and functional alterations in both ventricles.

## Introduction

Adult GH deficiency (GHD) is a heterogeneous disorder that may result from a variety of causes, including structural lesions, traumas, infiltrative diseases, surgery or irradiation to the pituitary gland and/or hypothalamus, or idiopathic dysfunction (1–5). Its diagnosis depends on the demonstration of a subnormal rise in peak serum GH level in response to one or more GH stimulation tests (1, 6–9). From a clinical point of view, this condition is characterized by many alterations, such as impaired quality of life, decreased lean mass, increased fat mass, abnormal lipid profile, osteoporosis, and increased all-cause mortality (10–13). The clinical relevance of cardiac involvement in patients with adult GHD remains, however, only partially elucidated.

Physiologically, the GH/IGF-I axis interacts with the cardiovascular system, both indirectly by acting on various metabolic cardiovascular risk factors, and directly by actively maintaining the structure and function of the normal adult heart, through the stimulation of cardiac growth and myocardial contractility (14–17). An impairment of the GH/IGF-I axis contributes to determine cardiovascular alterations, as suggested by clinical studies reporting an increase in cardiovascular morbidity and mortality in hypopituitary adults with untreated GHD (18–22). With respect to cardiac structure, adult patients with untreated GHD show a reduced left ventricular (LV) mass (LVM) and diameter, coherently accompanied by a reduction in LV wall thickness at the interventricular septum (IVS) and posterior wall (PW) (23–28). Data about the LV systolic and diastolic function are less clear; a reduction of LV systolic performance has been consistently observed mostly during peak exercise (23, 29–31); recently, a subclinical systolic disfunction by speckle-tracking echocardiography has been suggested (32), but further studies are needed to confirm this result; with respect to diastolic filling, an impairment has been suggested by some authors (24, 29), but not clearly confirmed throughout the literature (33, 34).

Concerning the cardiac effects of recombinant human GH (rhGH) replacement therapy in patients with adult GHD, a previous meta-analysis (33), based on 16 echocardiographic studies, found that rhGH therapy determined a significant increase in LVM (+10.8 g), IVS thickness (+0.28 mm), PW thickness (+0.98 mm), LV end-diastolic diameter (LVEDD, +1.34 mm) and LV stroke volume (LVSV, +10.3 ml). On the contrary, no significant effects were found on LV end-systolic diameter (LVESD) and fractional shortening (FS), which was used as a proxy for systolic function and ejection fraction (EF).

As the authors themselves acknowledge, a limitation of this analysis, as well as of most studies evaluating cardiac structure and function in adult patients with GHD, is represented by the use of echocardiography, which suffers from a relatively low reproducibility with respect to other imaging techniques and, in particular, compared to magnetic resonance imaging (MRI). Moreover, the included studies were characterized by a significant heterogeneity in patient populations, with remarkable age-differences, and commonly with the enrollment of both childhood-onset and adult-onset GHD patients. These limitations may account at least in part for the sometimes-discrepant results obtained by different studies; moreover, they may have impaired the potential to correctly characterize subtler cardiac abnormalities in patients with GHD. This is particularly relevant when dealing with the structure and function of the right ventricle (RV) (35, 36), whose echocardiographic assessment suffers from significant challenges and limitations (37); within the specific context of adult GHD, echocardiographic studies provide almost no data about possible alterations in RV morphology and function. As a consequence, whether or not adult GHD is characterized by a biventricular cardiac involvement still remains, *de facto*, unclear.

Compared to echocardiography, cardiac MRI represents a more reliable and reproducible technique for measuring cardiac volumes, mass, and function; its use in clinical research, given its enhanced precision and reproducibility, has been estimated to allow for a reduction in sample size of 80–95% to obtain equal statistical power compared to investigations based on conventional echocardiography (38). In fact, it avoids most of the geometrical assumption required by echocardiographic estimates, and is currently considered the gold standard for the assessment of cardiac morphology and functionality (38–40); notably, this is particularly true when evaluating the structure and function of the RV, as cardiac MRI is considered the most accurate and reproducible method for the assessment of RV parameters (35, 36, 41).

Given these premises, cardiac MRI might provide finer information about cardiac alterations in patients with adult GHD, as well as their possible changes after the initiation of rhGH replacement therapy; in particular, it might answer to the question about if and how RV is involved. Some studies have been published in this regard (42–45), with interesting results. However, the strength of their conclusions is hampered by their limited sample size, and a quantitative synthesis of their results is still lacking. The aim of this systematic review and meta-analysis was, thus, to specifically summarize and quantitatively combine the available evidence about the MRI assessment of cardiac function and morphology in patients with adult GHD.

## Methods

### Search Strategy and Study Selection

This study was conducted according to the Preferred Reporting Items for Systematic Reviews and Meta-Analysis (PRISMA) guidelines (46). The process of literature search and study selection was made by two independent reviewers (F.B., V.G.); all disparities were resolved through consensus.

The following electronic databases were queried until the February 1^st^ 2022: PubMed/Medline, EMBASE, Cochrane library, OVID, and CINAHL. The search strategy was performed using a combination of relevant database-specific search terms to identify pertinent studies about the evaluation of cardiac morphology and function by MRI in patients with adult GHD. The full search strategy is presented in Supplementary Material (**Appendix 1**). No filters were applied for study design, language, and publication date.

After duplicate removal, all studies found with the aforementioned search were evaluated for inclusion in the meta-analysis, first by title/abstract screening and then by full-text review. We excluded from our analysis studies according to the following exclusion criteria: (a) unavailability of any of the outcomes of interest, as defined in the following subsection; (b) case reports or case series; (c) conference abstracts. In case of patient overlap between studies, the one with the largest sample size was considered.

### Outcomes

The following outcomes were assessed: (a) comparison of left and right ventricular morphology and function, as assessed by cardiac MRI, between patients with GHD and controls; (b) comparison of left and right ventricular morphology and function, as assessed by cardiac MRI, before and after treatment with rhGH in patients with GHD.

More in detail, the MRI parameters that were evaluated were: (i) left ventricular ejection fraction (LVEF); (ii) left ventricular stroke volume index (LVSVi); (iii) left ventricular end-diastolic volume index (LVEDVi); (iv) left ventricular end-systolic volume index (LVESVi); (v) left ventricular mass index (LVMi); (vi) right ventricular ejection fraction (RVEF); (vii) right ventricular stroke volume index (RVSVi); (viii) right ventricular end-diastolic volume index (RVEDVi); (ix) right ventricular end-systolic volume index (RVESVi).

### Data Extraction

Two authors (F.B., V.G.) independently examined and extracted data from papers which met the inclusion criteria using pre-specified data extraction templates. For each eligible study, the following information were collected: (a) first author and publication year; (b) study design; (c) major selection criteria for each group; (d) matching criteria between GHD patients and controls; (e) number of subjects enrolled; (f) patients’ characteristics in terms of demographic data; (g) cardiac MRI data in GHD patients and controls, according to the parameters specified in the previous section; (h) cardiac MRI data in GHD patients before and after treatment with rhGH, according to the parameters specified in the previous section.

### Risk of Bias Assessment

The risk of bias was independently assessed for each included study by two authors (F.B., V.G.). The twenty components of the AXIS tool (Appraisal tool for Cross-Sectional Studies) (47) were used for the evaluation of cross-sectional studies comparing cardiac MRI parameters between patients with adult GHD and controls. The seven domains of the ROBINS-I tool (Risk Of Bias In Non-randomized Studies of Intervention) (48) were used for the evaluation of longitudinal studies evaluating the changes in cardiac MRI parameters before and after the initiation of rhGH therapy.

### Statistical Analysis

Continuous variables and categorical variables were reported as numbers and percentages, respectively. Comparisons between patients with GHD and controls were reported as mean difference and as standardized mean difference (SMD), expressed as Hedges’ g. Variations before and after rhGH treatment in patients with GHD were reported as mean paired differences. A random-effect restricted maximum likelihood model was adopted for statistical pooling of data. Higgins I^2^ statistics and Cochran Q test were used to assess heterogeneity between studies. Statistical analysis was performed using STATA 17 (StataCorp, College Station, Texas, USA).

## Results

### Search Results

**Figure 1:**
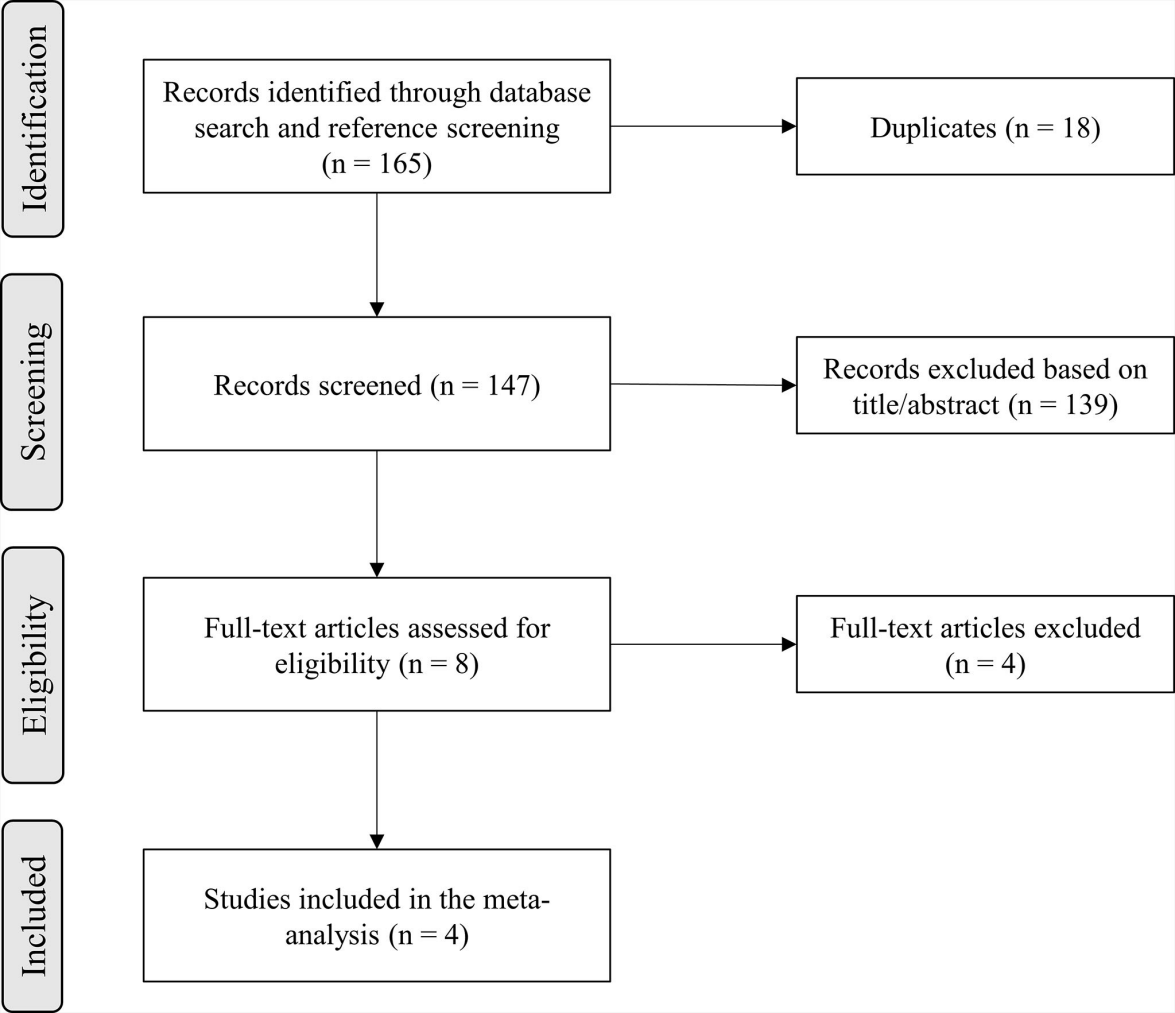
Flow-chart of study inclusion.

A total of 165 records were identified in the initial literature search. Removal of duplicates led to an overall pool of 147 studies. An accurate title or abstract revision was sufficient to exclude 139 articles as not pertinent or not fulfilling our prespecified inclusion or exclusion criteria. The remaining 8 studies were assessed in full-text for eligibility (42–45, 49–52), and 4 of them were excluded due to patient overlap (49–52); thus, 4 studies finally met all criteria for being included in the final analysis (42–45) (**Figure 1**).

### Characteristics of the Included Studies

**Table 1** summarizes the basic studies characteristics. Almost all included patients had an adult-onset GHD, with the only exception of two adult patients with a childhood-onset GHD enrolled in the study by Andreassen et al. (42). All studies provided data comparing GHD patients with controls, in a cross-sectional design (42–45). The matching criteria adopted for the selection of the control group included age and sex in all studies (42–45); body surface area (BSA) was considered as an adjunctive matching criterion in two studies (42, 43), and body mass index (BMI) in one (43). Moreover, three out of four studies also provided a longitudinal evaluation of the effect of rhGH treatment on cardiac MRI parameters in the GHD group (42, 44, 45). Two of them had an observational design (42, 45), while one was designed as a randomized controlled trial with an open-label extension (44).

**Table 1:** Study characteristics.

First author, year	Study design	Matching criteria between patients with GHD and controls	N of subjects^a^	Gender distribution^a^(% male)	Mean age^a^ (years)	Tests and cut-offs used for the definition of GHD	Duration of rhGH treatment (months)
Andreassen et al., 2011 (42)	Cross-sectional + Observational	Age, sex, BSA	16^b^/16	50.0/50.0	49.0/49.0	GHRH + PD GH cut-off: < 6.5 ng/ml	12
De Cobelli et al., 2019 (43)	Cross-sectional	Age, sex, BSA, BMI	15/15	53.3/53.3	52.0/49.0	GHRH + ARG GH cut-off: BMI-dependent^c^	NA
Gonzalez et al., 2017 (44)	Cross-sectional + RCT + OLE	Age, sex	17^d^/16	58.8/56.3	48.4/NA	ITT GH cut-off: < 3 ng/ml	12^e^
Thomas et al., 2016 (45)	Cross-sectional + Observational	Age, sex	10^f^/10	80.0/80.0	55.0/54.0	ITT or GST GH cut-off: < 3 ng/ml	12

a Data regarding the GHD patient group and the control group are reported in this order, separated by a slash.

b Two patients were excluded from longitudinal analyses because they were lost at follow-up.

c < 11.5 ng/ml for lean subjects, < 8.0 ng/ml for overweight subjects, < 4.2 ng/ml for obese subjects.

d One patient was excluded because he had an intracranial clip and could not undergo cardiac MRI; one patient was excluded because he developed GHD after being cured for acromegaly.

e 6-month cross-over RCT, followed by a 6-month OLE.

f One patient was excluded because he was discovered to have hypertension and diabetes; his age- and sex-matched control subjects was excluded as well; two more patients were excluded from longitudinal analyses because they were lost at follow-up.

ARG, arginine; BMI, body mass index; BSA, body surface area; GH, growth hormone; GHD, growth hormone deficiency; GHRH, growth hormone releasing hormone; GST, glucagon stimulation test; ITT, insulin tolerance test; N, number; NA, not applicable/not available; OLE, open-label extension; PD, pyridostigmine; RCT, randomized controlled trial; rhGH, recombinant human growth hormone.

Of note, in the study by Thomas et al. (45), all parameters were declared to be summarized using standard deviation (SD) as the index of dispersion, but the reported p-values and interpretation of the results were internally consistent with the data only if the reported index of dispersion was actually the standard error (SE); thus, in our analysis, this erratum was taken into account, and SE were transformed into SD before our quantitative analyses.

### Comparison of Cardiac MRI Parameters Between Patients With GHD and Controls

Data about LVEF (42–45) and LVMi (42–45) were reported in all studies; data about LVEDVi (42, 43, 45) and LVESVi (42, 43, 45) were reported in three studies; data about LVSVi (42, 45), RVEF (43, 45), RVEDVi (43, 45) and RVESVi (43, 45) were reported in two studies; data about RVSVi (45) were reported in one study.

**Figure 2:**
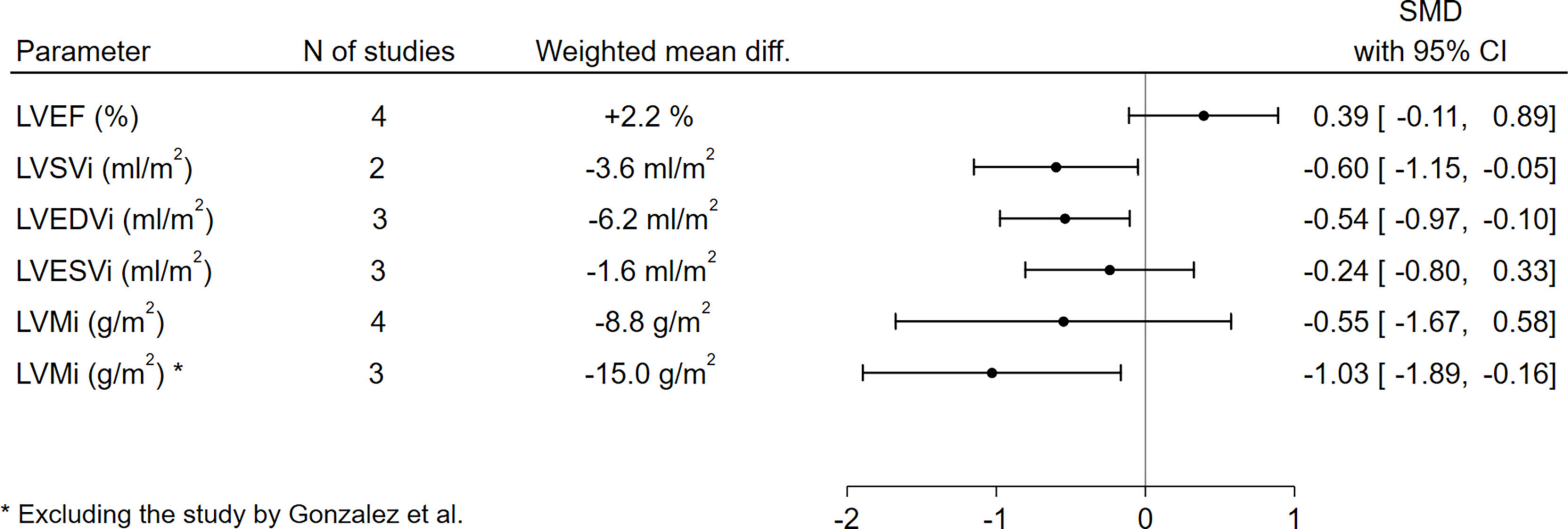
Comparison of LV functional and morphological parameters between patients with GHD and controls. CI, confidence interval; GHD, growth hormone deficiency; LV, left ventricle; LVEDVi, left ventricular end-diastolic volume index; LVEF, left ventricular ejection fraction; LVESVi, left ventricular end-systolic volume index; LVMi, left ventricular mass index; LVSVi, left ventricular stroke volume index; N, number; SMD, standardized mean difference.

With respect to LV function and morphology, LVSVi (-3.6 ml/m^2^, SMD -0.60, 95%CI [-1.15,-0.05], p=0.03) and LVEDVi (-6.2 ml/m^2^, SMD -0.54, 95%CI [-0.97,-0.10], p=0.02) were significantly lower in GHD patients compared to controls. On the other hand, no significant differences between GHD patients and controls could be found in terms of LVEF (+2.2%, SMD 0.39, 95%CI [-0.11,0.89], p=0.13) or LVESVi (-1.6 ml/m^2^, SMD -0.24, 95%CI [-0.80,0.33], p=0.41). When assessing LVMi, no overall differences could be found when pooling data from all studies (-8.8 g/m^2^, SMD -0.55, 95%CI [-1.67,0.58], p=0.34); this result, however, was remarkably influenced by the findings by Gonzalez et al. (44), as GHD patients presented a high rate of poorly controlled hypertension, which – as the author themselves recognize – could have significantly biased the assessment of LVMi in this cohort; excluding this paper from the analysis, the pooled effect sizes would yield significantly lower LVMi values in GHD patients compared to controls (-15.0 g/m^2^, SMD -1.03, 95%CI [-1.89,-0.16], p=0.02) (**Figure 2** and **Supplementary Figure 1**).

**Figure 3:**
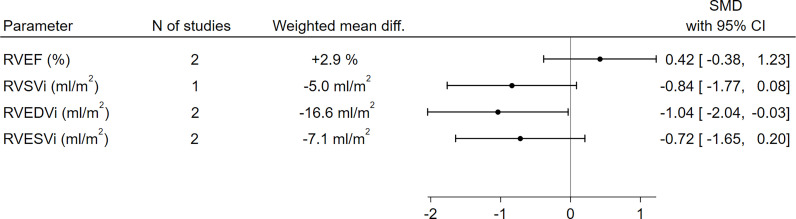
Comparison of RV functional and morphological parameters between patients with GHD and controls. CI, confidence interval; GHD, growth hormone deficiency; RV, right ventricle; RVEDVi, right ventricular end-diastolic volume index; RVEF, right ventricular ejection fraction; RVESVi, right ventricular end-systolic volume index; RVSVi, right ventricular stroke volume index; N, number; SMD, standardized mean difference.

With respect to RV function and morphology, RVEDVi (-16.6 ml/m^2^, SMD -1.04, 95%CI [-2.04,-0.03], p=0.04) was significantly lower in GHD patients compared to controls, and a borderline-significant trend towards a lower RVSVi (-5.0 ml/m^2^, SMD -0.84, 95%CI [-1.77,0.08], p=0.07) could also be observed. On the other hand, no significant differences between GHD patients and controls could be found in terms of RVEF (+2.9%, SMD 0.42, 95%CI [-0.38,1.23], p=0.30) or RVESVi (-7.1 ml/m^2^, SMD -0.72, 95%CI [-1.65,0.20], p=0.13) (**Figure 3** and **Supplementary Figure 2**).

### Variation of Cardiac MRI Parameters Before and After rhGH Treatment in Patients With GHD

Data concerning the variation of cardiac MRI parameters after rhGH treatment initiation in patients with GHD were sparser. Moreover, to the scope of the present meta-analysis, this evaluation was further limited by the unavailability, in most cases, of the exact data summarizing the paired differences of the parameters between baseline and follow-up, which would have been necessary for a correct quantitative synthesis of the results given the paired design of the research question.

**Figure 4:**
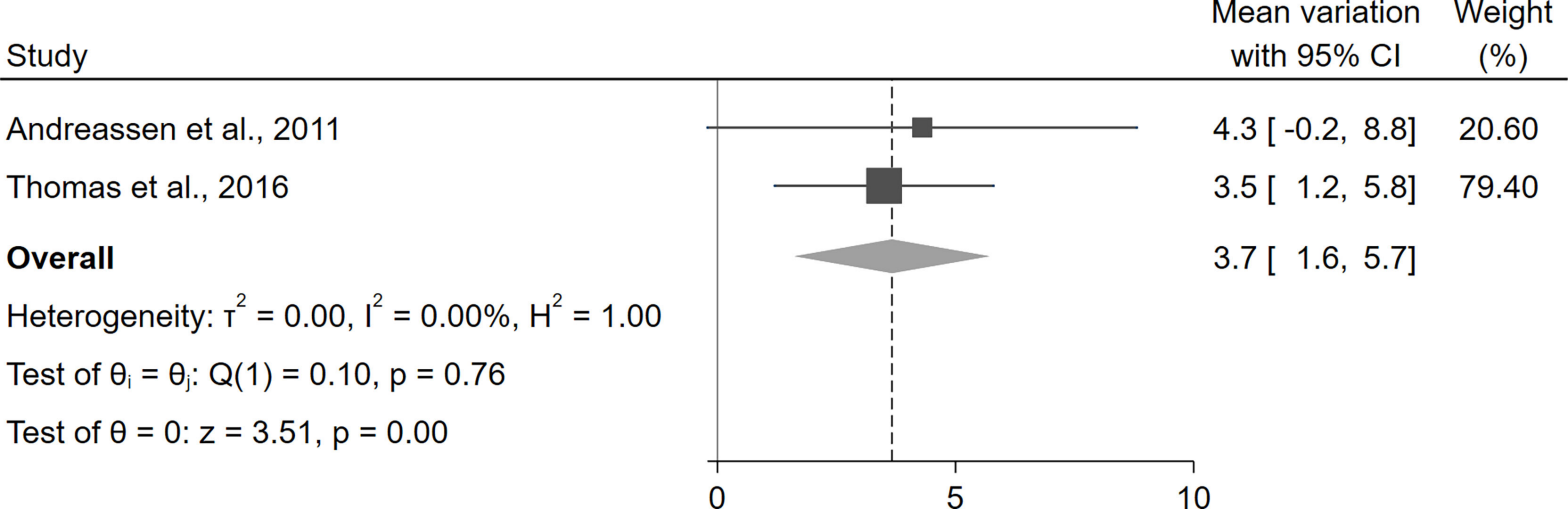
Variation of LVMi before and after treatment with rhGH in patients with GHD. CI, confidence interval; GHD, growth hormone deficiency; LVMi, left ventricular mass index; rhGH, recombinant human growth hormone.

Overall, the only parameter for which exact data about the variation between baseline and follow-up were available was LVMi, which was reported in one study (42) and obtainable by the supplementary material in another one (45). The statistical pooling of these results suggested a statistically significant increase in LVMi after the initiation of rhGH therapy (+3.7 g/m^2^, 95%CI [1.6,5.7], p<0.01) (**Figure 4**). For all other parameters, the considered manuscripts only reported the pooled means at baseline and at the study end, without providing the mean paired differences and thus preventing a quantitative synthesis of these results. Nevertheless, as a qualitative appraisal, no significant variation in any parameter was found.

### Quality Assessment

The results of the quality assessment of the studies are reported in **Supplementary Tables 1, 2**. Altogether, the risk of bias appeared to be moderate-to-low in most studies, with the exception of a significant concern related to the likely presence of confounding and selection bias in the study by Gonzalez et al. (44), as more specifically discussed in other sections of our paper. Publication bias was not formally assessed due to the low number of available studies.

## Discussion

This is the first systematic review and meta-analysis specifically evaluating the available evidence about the assessment by MRI of cardiac function and morphology in adult patients with GHD.

With respect to cardiac morphology, the available evidence pointed out a relevant impact of adult GHD on ventricular volumes. The LVEDVi and RVEDVi were significantly lower in GHD patients compared to controls; on the other hand, the impact of GHD on end-systolic volumes seemed to be less evident, as no significant differences between patients with GHD and controls could be found neither for the left nor for the right ventricle. Coherently with these data, the stroke volume, which can be calculated as the difference between the end-diastolic volume and the end-systolic volume, was found to be significantly lower in patients with GHD for the left ventricle, with a borderline-significance also for the right ventricle.

When considering systolic function, no significant differences could be found in terms of EF between GHD patients and controls, neither for the left nor for the right ventricle. Overall, these findings were in line with those already discussed about the left and right ventricular volumes; in fact, the EF can be computed as the ratio between the stroke volume and the end-diastolic volume, which were found to be both decreased (either significantly or with a borderline-significant trend) in GHD patients compared to control. Overall, thus, the data about the EF demonstrate that, in each ventricle, the reduction in stroke volume and end-diastolic volume was roughly proportional, with an overall maintenance of the fraction of ventricular blood that is pumped into the pulmonary or systemic arterial system at each stroke.

With respect to cardiac mass, the available data were contrasting. Physiologically, the somatotroph axis is known to exert a direct anabolic function on the cardiac muscle, as widely demonstrated, for example, in experimental models (53–55) and in patients with acromegaly (23, 24, 56–58); moreover, especially in males, an indirect anabolic effect *via* the hypothalamic-pituitary-gonadal axis is also possible, given the known cross-talk between the two axes (59) and the recognized anabolic effects of testosterone on cardiomyocytes (60). Previous data based on echocardiographic studies suggested that GHD patients were characterized by a reduced LV diameter and mass, accompanied by a reduction in LV wall thickness at the IVS and PW (23–28). Moreover, a meta-analysis, also based on echocardiographic studies, showed that the initiation of rhGH replacement therapy was associated with an increase in LV mass, IVS wall thickness, and PW thickness (33). In the present meta-analysis, when pooling the retrieved data on LV mass, a significant heterogeneity was found, mostly driven by a study by Gonzalez et al. (44), in which – contrarily to the common knowledge about the pathophysiological effect of GHD on cardiac structure – patients with GHD were found to have an increased LV mass compared to controls. However, as we already pointed out in the Results section of the present manuscript, this finding suffered from a remarkable bias given by the high rate of GHD patients with a poorly controlled hypertension; these patients, in fact, showed significantly higher systolic blood pressure values with respect to the control group (143 mmHg vs. 131 mmHg), with a reported mean systolic value above what is considered appropriate as an adequate target for blood pressure control. This could result in a significant bias on the retrieved results, as arterial hypertension, especially if poorly controlled, is a widely known stimulus for an increase in LV mass and, ultimately, a risk factor for the development of LV hypertrophy (61–63). When excluding this study from the analysis, the statistical pooling of the results retrieved by the other three studies unequivocally showed a significantly lower LV mass in GHD patients, which was consistent with previous findings by echocardiographic studies and, more broadly, with the well-established pathophysiology of the disease. When shifting the focus on the effect of rhGH replacement therapy on cardiac mass, the available data, as previously stated, are sparse; nevertheless, our findings were in line with those previously found by echocardiographic studies (33), showing an increase in LVMi after rhGH treatment initiation.

The main strength of this meta-analysis is the selection of studies assessing cardiac morphology and function by MRI, which is currently considered the gold-standard imaging technique to this scope. In fact, with respect to echocardiography, it is endowed with a higher accuracy and reproducibility, reducing the risk of biases, and possibly improving the potential to correctly characterize subtler cardiac abnormalities in patients with GHD. This is of utmost relevance when examining RV parameters; in fact, the echocardiographic assessment of the RV suffers from significant challenges and limitations and, within the specific context of adult GHD, available echocardiographic studies provided almost no data about possible alterations in RV morphology and function.

Our meta-analysis had also some limitations. First, the strength of the conclusions was limited by the small number of available studies; this limitation could reasonably be expected, in light of the low frequency of GHD together with the relatively limited availability of cardiac MRI; on the other hand, as already pointed out, cardiac MRI has the significant advantage of a better precision and reproducibility of estimates compared to echocardiography, and it has been estimated that a sample size reduced by 80–95% is still sufficient to obtain equal statistical power compared to echocardiographic studies (38). Second, the quality of the results was limited by that of the included studies; however, the risk of bias was generally moderate-to-low, except for the study by Gonzalez et al. (44), whose potential biases have been already discussed and taken into account in the quantitative analyses. Third, patients’ characteristics and inclusion criteria could differ between studies in some aspects, such as the underlying pituitary disease, the severity and presumed duration of GHD, and the diagnostic tests adopted for its definition, among others; this could be responsible for a certain degree of heterogeneity in the considered outcomes; nevertheless, heterogeneity is a common limitation of all meta-analyses, and appropriate statistical methods – such as the use of a random-effect model – were adopted to account for it. Fourth, the comparisons of cardiac MRI parameters between groups were all based on crude differences, as derived by univariate analyses; thus, the possible interplay with other predictors could not be evaluated. Fifth, no data were available about the effects of rhGH replacement therapy on cardiac function and morphology beyond the first year; therefore, the long-term course of cardiac MRI parameters during prolonged treatment with rhGH still remains to be elucidated.

## Conclusions

In conclusion, the available evidence provided by cardiac MRI studies highlights significant left ventricular changes in patients with adult GHD, which are resumable as a reduction in end-diastolic volume, stroke volume, and ventricular mass; moreover, a significant increase in left ventricular mass can be seen after the initiation of rhGH replacement therapy. In addition, this is the first meta-analysis to provide a quantitative evaluation of the right ventricular involvement in GHD patients, for which echocardiographic data are nearly absent. Our results suggest a pattern of right ventricular alterations which is similar to left ventricular ones, with an almost significant reduction in end-diastolic volume and a statistical trend towards a lower stroke volume. This provides relevant information supporting a biventricular cardiac involvement in GHD, overall characterized by similar changes in left and right ventricular volumes and function.

## Data Availability Statement

The original contributions presented in the study are included in the article/**Supplementary Material**. Further inquiries can be directed to the corresponding author.

## Author Contributions

FB contributed to work conceptualization, data collection, data analysis and manuscript writing. NP, AB, CB, and MP-C contributed to data interpretation and manuscript writing. RF, SG, and EG supervised the manuscript drafting. VG contributed to work conceptualization, data collection, data analysis, manuscript writing and final draft supervision. All authors approved the manuscript in its final form.

## Conflict of Interest

The authors declare that the research was conducted in the absence of any commercial or financial relationships that could be construed as a potential conflict of interest.

## Publisher’s Note

All claims expressed in this article are solely those of the authors and do not necessarily represent those of their affiliated organizations, or those of the publisher, the editors and the reviewers. Any product that may be evaluated in this article, or claim that may be made by its manufacturer, is not guaranteed or endorsed by the publisher.
