# Supplementary material for: MRI Assessment of Cardiac Function and Morphology in Adult Patients With Growth Hormone Deficiency: A Systematic Review and Meta-Analysis
Source: Front Endocrinol (Lausanne). 2022 Jun 10;13:910575. doi: 10.3389/fendo.2022.910575 (PMC9226436; doi:10.3389/fendo.2022.910575)
Supplement: Supplementary file 1 [file DataSheet_1.pdf]

Supplementary Material

Supplementary Table 1. Quality assessment of cross-sectional studies according to AXIS tool.

	Andreassen et al, 2011 (42)	De Cobelli et al, 2019 (43)	Gonzalez et al, 2017 (44)	Thomas et al, 2016 (45)
1. Were the aims/objectives of the study clear?	Y	Y	Y	Y
2. Was the study design appropriate for the stated aim (s)?	Y	Y	Y	Y
3. Was the sample size justified?	Y	N	Y	N
4. Was the target/reference population clearly defined? (Is it clear who the research was about?)	Y	Y	Y	Y
5. Was the sample frame taken from an appropriate population base so that it closely represented the target/reference population under investigation?	Y	Y	Y	Y
6. Was the selection process likely to select subjects/participants that were representative of the target/reference population under investigation?	Y	Y	Y	Y
7. Were measures undertaken to address and categorise non-responders?	N	N	N	N
8. Were the risk factor and outcome variables measured appropriate to the aims of the study?	Y	Y	Y	Y
9. Were the risk factor and outcome variables measured correctly using instruments/ measurements that had been trialled, piloted or published previously?	Y	Y	Y	Y

10. Is it clear what was used to determined statistical significance and/or precision estimates? (eg, p values, confidence intervals)	Y	Y	Y	Y
11. Were the methods (including statistical methods) sufficiently described to enable them to be repeated?	Y	Y	Y	Y
12. Were the basic data adequately described?	Y	Y	Y	Y
13. Does the response rate raise concerns about non-response bias?	N	N	N	N
14. If appropriate, was information about non-responders described?	N	N	N	N
15. Were the results internally consistent?	Y	Y	Y	N
16. Were the results for the analyses described in the methods presented?	Y	Y	Y	Y
17. Were the authors' discussions and conclusions justified by the results?	Y	Y	Y	Y
18. Were the limitations of the study discussed?	Y	Y	Y	Y
19. Were there any funding sources or conflicts of interest that may affect the authors' interpretation of the results?	N	N	N	N
20. Was ethical approval or consent of participants attained?	Y	Y	Y	Y

Abbreviations: N, no; Y, yes; U, unknown/unclear.

Supplementary Table 2. Quality assessment of longitudinal studies according to ROBINS-I tool.

	Andreassen et al, 2011 (42)	Gonzalez et al, 2017 (44)	Thomas et al, 2016 (45)
Bias due to confounding	Low	High	Low
Bias in selection of participants into the study	Low	Moderate	Low
Bias in classification of interventions	Low	Low	Low
Bias due to deviations from intended interventions	Low	Low	Low
Bias due to missing data	Moderate	Moderate	Moderate
Bias in measurement of outcomes	Low	Low	Low
Bias in selection of the reported result	Low	Low	Low
Overall bias	Moderate	High	Moderate

Supplementary Figure 1. One-by-one comparisons of LV functional and morphological parameters between patients with GHD and controls. In details: LVEF (A); LVSVi (B); LVEDVi (C); LVESVi (D): LVMi (E); LVMi with the exclusion of the study by Gonzalez et al (44) (F).

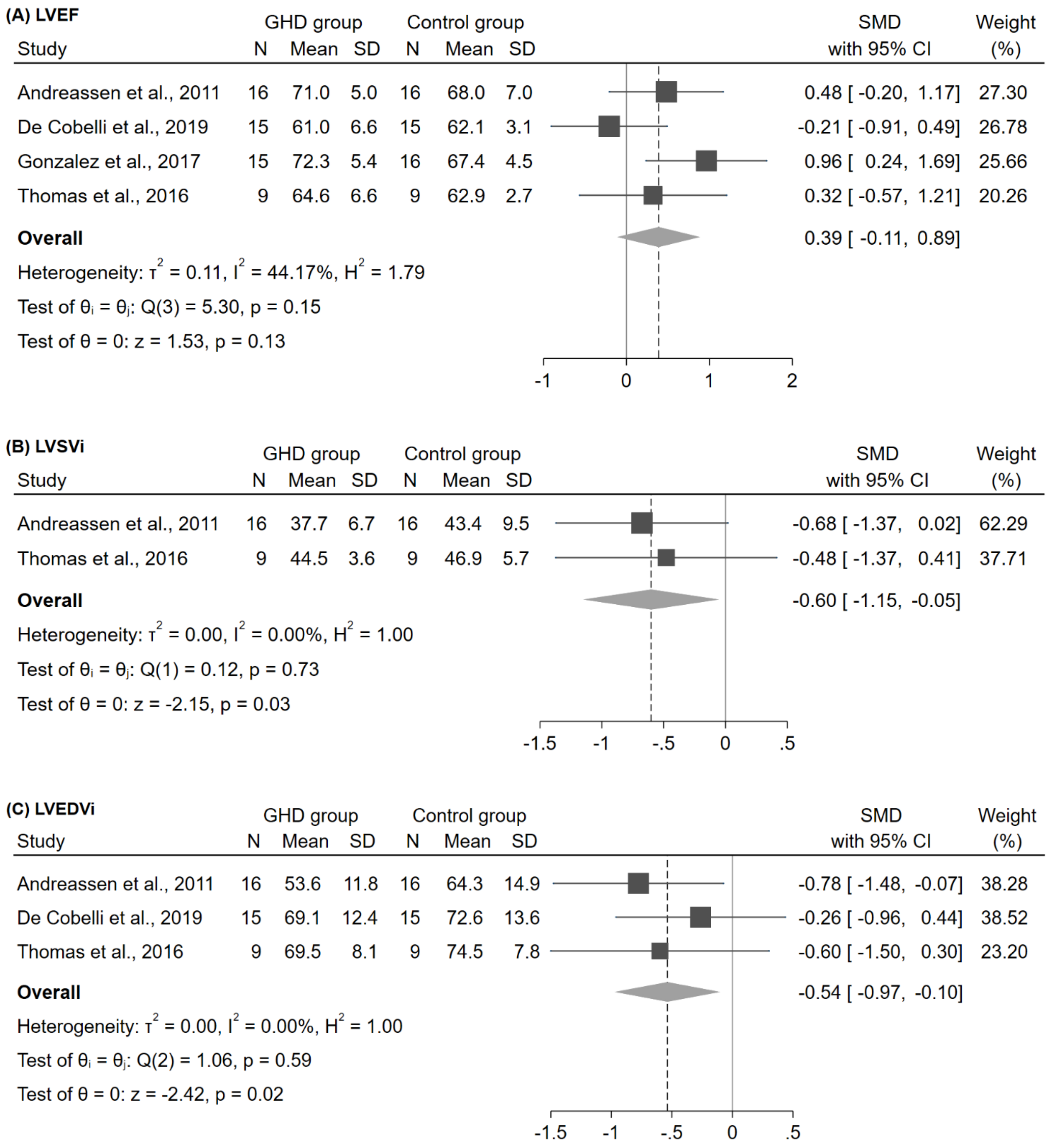

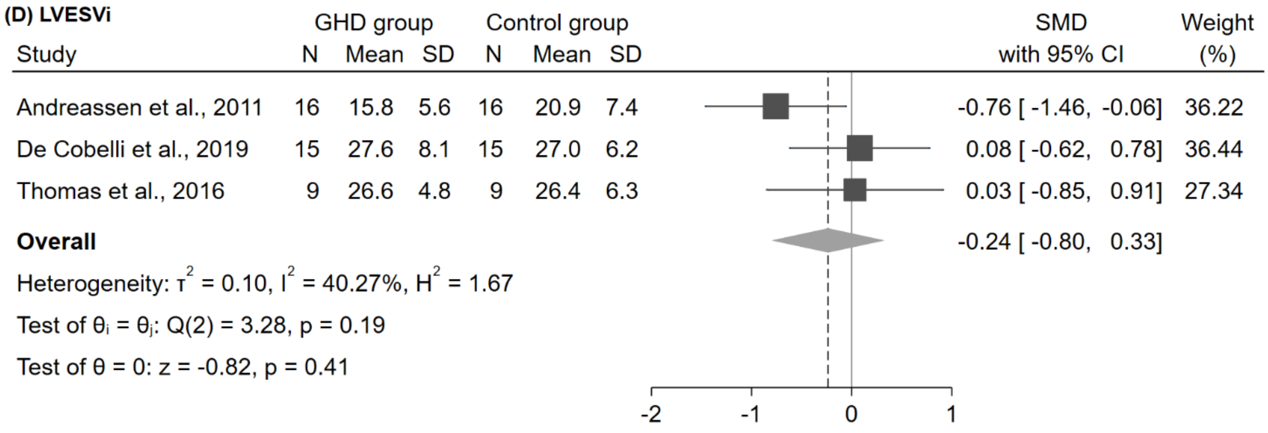

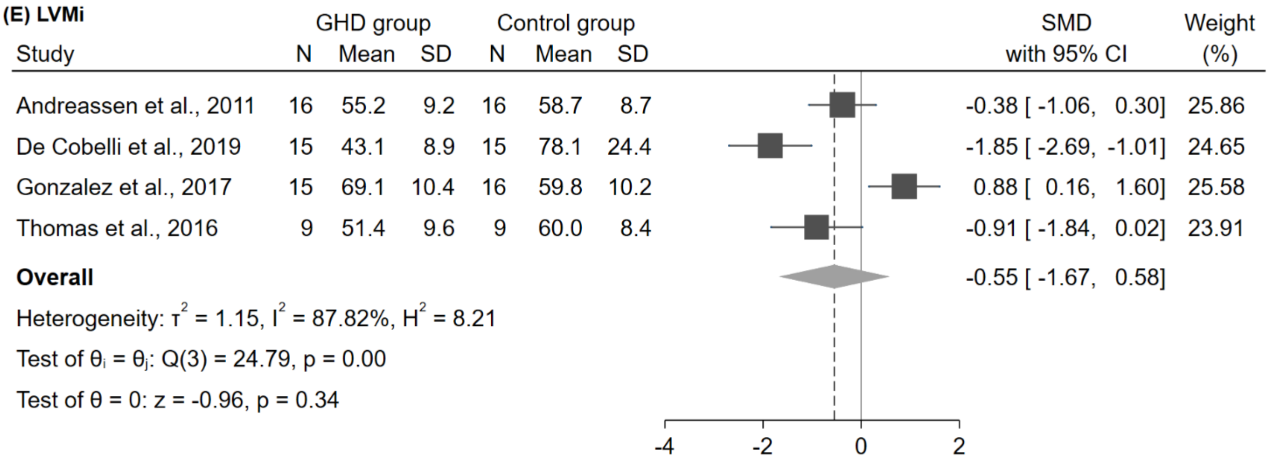

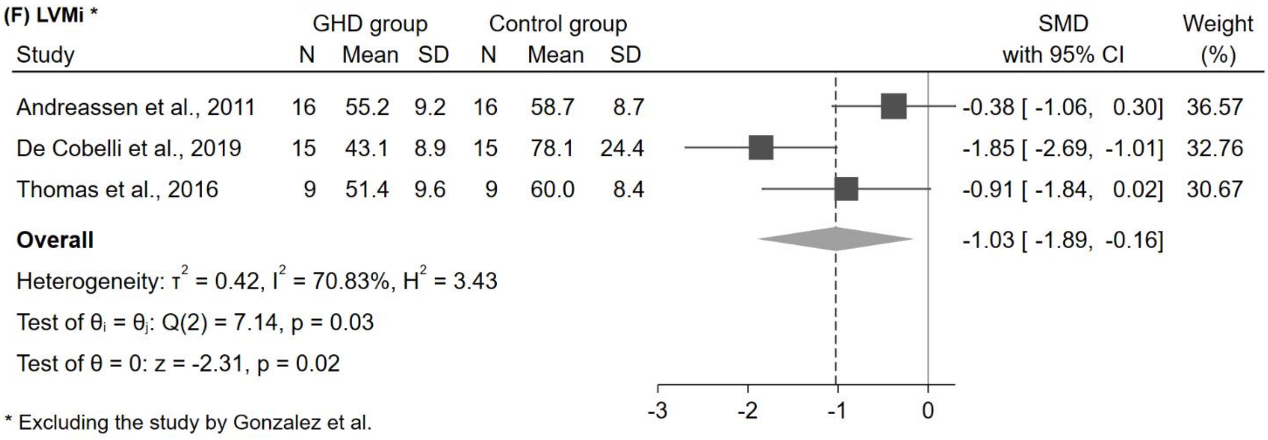

Abbreviations: CI, confidence interval; GHD, growth hormone deficiency; LV, left ventricle; LVEDVi, left ventricular end-diastolic volume index; LVEF, left ventricular ejection fraction; LVESVi, left ventricular end-systolic volume index; LVMi, left ventricular mass index; LVSVi, left ventricular stroke volume index; N, number; SD, standard deviation; SMD, standardized mean difference.

Supplementary Figure 2. One-by-one comparisons of RV functional and morphological parameters between patients with GHD and controls. In details: RVEF (A); RVSVi (B); RVEDVi (C); RVESVi (D).

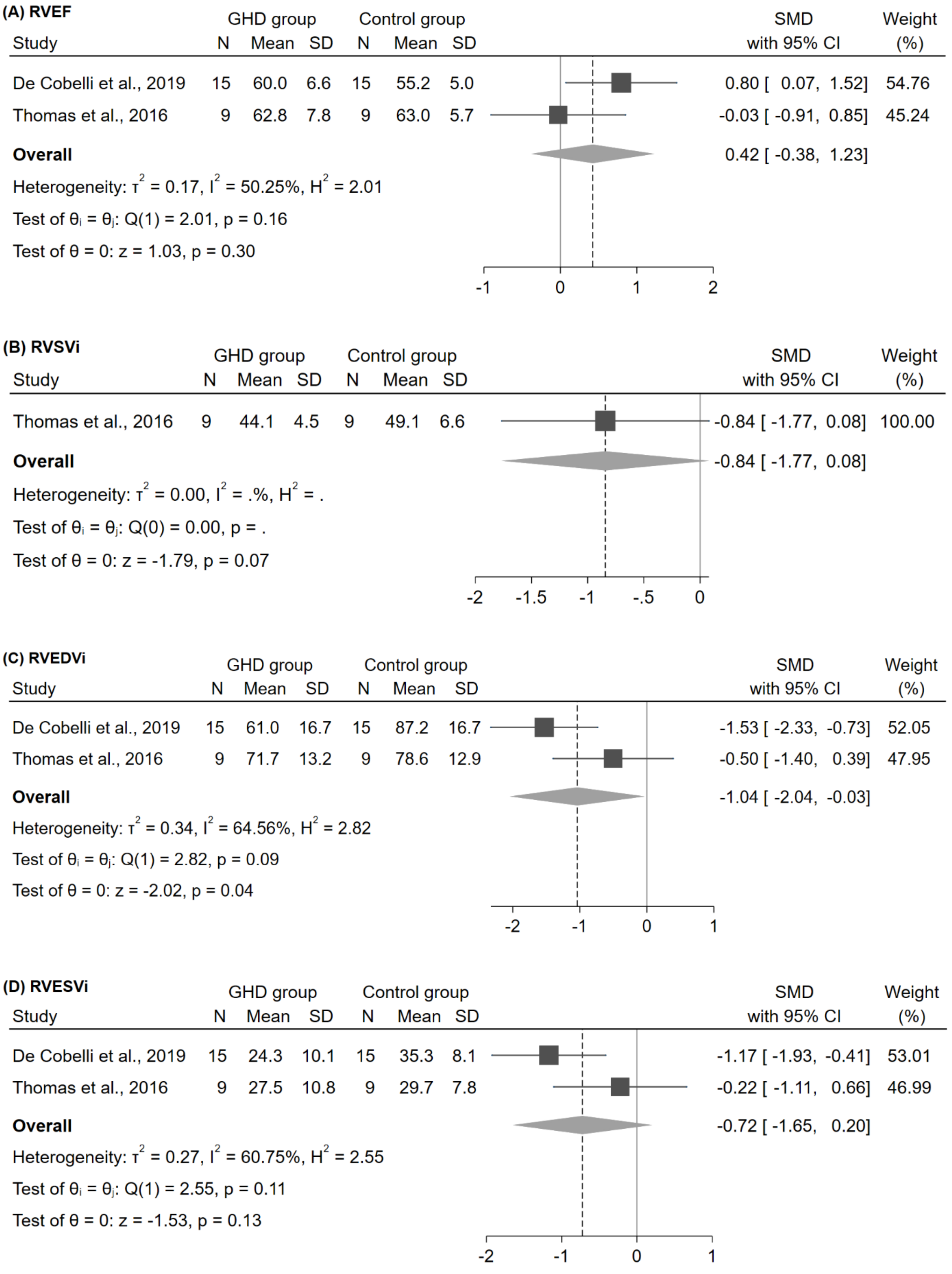

Abbreviations: CI, confidence interval; GHD, growth hormone deficiency; RV, right ventricle; RVEDVi, right ventricular end-diastolic volume index; RVEF, right ventricular ejection fraction; RVESVi, right ventricular end-systolic volume index; RVSVi, right ventricular stroke volume index; N, number; SD, standard deviation; SMD, standardized mean difference.

Appendix 1. Electronic search strategy.

**PUBMED**<br>*Advanced search, All fields*
("GHD" OR "GH deficiency" OR "growth hormone deficiency") AND ("cardiac magnetic resonance" OR "cardiac magnetic resonance imaging" OR "cardiac MRI" OR "CMR")

**EMBASE**<br>*Quick search, All fields*
("GHD" OR "GH deficiency" OR "growth hormone deficiency") AND ("cardiac magnetic resonance" OR "cardiac magnetic resonance imaging" OR "cardiac MRI" OR "CMR")

**COCHRANE LIBRARY**<br>*Advanced search, All text*
("GHD" OR "GH deficiency" OR "growth hormone deficiency") AND ("cardiac magnetic resonance" OR "cardiac magnetic resonance imaging" OR "cardiac MRI" OR "CMR")

**OVID**<br>*All resources, Multi-field search, All fields*
("GHD" OR "GH deficiency" OR "growth hormone deficiency") AND ("cardiac magnetic resonance" OR "cardiac magnetic resonance imaging" OR "cardiac MRI" OR "CMR")

**CINAHL**<br>*Advanced search, No field selected*
("GHD" OR "GH deficiency" OR "growth hormone deficiency") AND ("cardiac magnetic resonance" OR "cardiac magnetic resonance imaging" OR "cardiac MRI" OR "CMR")
